# Experiences of health care providers on pregnancy and childbirth care during the COVID-19 pandemic in Iran: a phenomenological study

**DOI:** 10.1186/s12884-021-04148-y

**Published:** 2021-10-03

**Authors:** Sedigheh Hantoushzadeh, Maryam Bagheri, Marjan Akhavan Amjadi, Maryam Farmahini Farahani, Fedyeh Haghollahi

**Affiliations:** 1 Vali Asr Reproductive Health Research Center, Tehran University of Medical Sciences, Tehran, Iran; 2 Department of Midwifery, Faculty of Nursing and Midwifery, Tehran Medical Science, Islamic Azad University, Tehran, Iran

**Keywords:** Health care providers, Experience, Pregnancy and childbirth care, COVID-19 pandemic

## Abstract

**Background:**

Coronavirus currently cause a lot of pressure on the health system. Accordingly, many changes occurred in the way of providing health care, including pregnancy and childbirth care. To our knowledge, no studies on experiences of maternity care Providers during the COVID-19 Pandemic have been published in Iran. We aimed to discover their experiences on pregnancy and childbirth care during the current COVID-19 pandemic.

**Methods:**

This study was a qualitative research performed with a descriptive phenomenological approach. The used sampling method was purposive sampling by taking the maximum variation possible into account, which continued until data saturation. Accordingly, in-depth and semi-structured interviews were conducted by including 12 participants, as 4 gynecologists, 6 midwives working in the hospitals and private offices, and 2 midwives working in the health centers.

Data were analyzed using Colaizzi’s seven stage method with MAXQDA10 software.

**Results:**

Data analysis led to the extraction of 3 themes, 9 categories, and 25 subcategories. The themes were as follows: “Fear of Disease”, “Burnout”, and “Lessons Learned from the COVID-19 Pandemic”, respectively.

**Conclusions:**

Maternal health care providers experience emotional and psychological stress and work challenges during the current COVID-19 pandemic. Therefore, comprehensive support should be provided for the protection of their physical and mental health statuses. By working as a team, utilizing the capacity of telemedicine to care and follow up mothers, and providing maternity care at home, some emerged challenges to maternal care services can be overcome.

## Background

COVID-19 is considered as a life-threatening respiratory disease that has rapidly spread all around the world and then caused serious public health problems [1]. It was estimated that one-third to two-thirds of the world’s population are likely to be infected with this virus. Accordingly, its Case-fatality rate is about 2.3% [2, 3]. This disease is more severe in men, older people, and those with underlying diseases such as cardiovascular diseases and diabetes, and causes more morbidity and mortality [4]. Some studies are evaluating the effect of pregnancy as a risk factor for the consequences of this disease [5].

There are more than 100 million pregnant mothers worldwide; therefore, in fact all of them currently are at risk for the disease [2]. During pregnancy, the manifestations of respiratory viral diseases due to physiological changes in the immune and cardiopulmonary systems, are usually more severe [1]. SARS and *MERS* respiratory syndromes belong to the coronavirus family chain, which could cause severe pulmonary complications and even death during pregnancy [6, 7]. Since pneumonia is the most common non-obstetric infection and responsible for many indirect maternal deaths, so any viral infection leading to respiratory infection during pregnancy is life-threatening for the pregnant mothers and requires immediate attention during pregnancy [8]. Besides maternal outcomes of COVID-19, the possibility of fetal effects and neonatal outcomes can also be considered [9].

The results of several studies on the effects of the COVID-19 pandemic on maternal and perinatal outcomes showed the increased risks of preterm delivery, cesarean section, ruptured ectopic pregnancies, maternal depression, still birth, and needing admission to the neonatal intensive care unit. Correspondingly, these complications are exacerbated if the mother develops pneumonia and respiratory complications and needs hospitalization [2, 10–12].

Currently, most of the available clinical data on the effects of COVID-19 on pregnancy are often limited only to cases of late-onset maternal infection, and no information are available on the consequences of early-onset pregnancy [9]. Some countries like the United Kingdom, consider pregnant mothers as members of the vulnerable group, so they consider cautious principles in their care [5].

Coronavirus has a very high infectiousness rate; therefore, it has put a lot of pressure on the health care system. The results of preliminary studies have reported the incidence rate of COVID- 19 in health workers between 3.8 and 63% [3]. The lack of medical facilities, staff shortages, and unpredictable nature of the disease as well as widespread transmission of the virus were found to have severe consequences for health system in more than 200 countries [13]. Health care workers who had no experience of working with infectious diseases, experienced a lot of stress under this condition [14]. The results of the Lai et al’s study (2020) showed that a significant percentage of physicians and nurses experienced depressive, anxious, and distressing symptoms during the current COVID-19 pandemic [15].

As well, there have been many changes in the way of providing health care. Accordingly, these changes include social distancing measures, delaying unnecessary surgeries, avoiding unnecessary referrals, and performing some health care virtually [16]. Pregnancy and childbirth care have also changed significantly under this condition. During the COVID-19 pandemic, maternal health care providers, who are at the frontline of caring for pregnant women and infants, have to change the way of caring, in order to prevent the spread of the virus. Moreover, they should provide family-centered care and have a good relationship with mothers to support them emotionally and to manage their stress. Additionally, with the reduced number of prenatal visits, they should make practical decisions on choosing the safest way to provide quality care, while they should also be adapted to changes in guidelines and resource constraints [17].

Considering the importance of the role of maternity care providers in maternal health during pregnancy and childbirth as well as the unknown nature of this disease and its possible effects on both pregnancy and childbirth, conducting a study to discover the experiences of health care provider about pregnancy and childbirth care during the COVID-19 pandemic seems to be essential.

The levels of readiness and responsiveness in maternity care units are quite different between various institutions and countries, and currently no study has been conducted to discover experiences of health care provider about maternity care during the COVID-19 pandemic in Iran yet. Clearly, recognizing different views and experiences of health care providers regarding pregnancy and childbirth care during the pandemic of COVID-19, will lead to the discovery of some challenges in this field and how to be adapted with them, which can lead to effective responses to quality care.

Qualitative studies with the unique feature of in-depth description of phenomena can be used to describe the experiences of health care workers from life, events, and trends [18]. Therefore, this qualitative study aimed to understand the experiences of health care provider from maternity care during the COVID-19 pandemic.

## Methods

### Study design

The present study was a qualitative research performed using a descriptive phenomenological approach. This method was used to answer the research question “What are the challenges and adaptive strategies in pregnancy and childbirth care in the COVID-19 pandemic from the perspective of health care providers?”

### Participants

The sampling method was snowball and purposive sampling with taking the maximum variation possible into account in order to obtain different opinions on the phenomenon from the best informants [19]. Accordingly, midwives and gynecologists working in health centers, hospitals or private offices in Tehran who had at least 3 years of work experience and were responsible for caring pregnant women during the COVID-19 pandemic, were selected to be included in this study. Sampling continued until data saturation, i.e. until no new information was obtained.

### Data collection

Simultaneous collection and analysis of qualitative data were done from June to December, 2020. For data collection, in-depth semi-structured individual interviews were conducted. The researcher conducted these interviews after obtaining the ethical confirmation from the Ethical Committee of Tehran University of Medical Sciences for this study. Before conducting each interview, the researcher provided participants with information on the nature and purpose of the study and the confidentiality of their conversations, and then received written consent from them to participate in the study. Notably, their voices in each interview were recorded. The participants’ personal information such as age, level of education, profession, work experience, and place of employment were collected by a questionnaire. Thereafter, they were asked to share their experiences with pregnancy and childbirth care during the COVID-19 pandemic. The interview began by asking guiding questions and then continued by asking exploratory questions. Open-ended follow-up questions were used to obtain detailed descriptions. Some of the guiding questions were as follows:

“Please tell me about your experience on Pregnancy and Childbirth Care during the current situation (The COVID-19 pandemic).”

“What is the difference between providing care during the COVID-19 pandemic and before?”

“How did you feel in the early days of the COVID-19 pandemic?”

“How do you feel now?”

“What challenges did you face? How did you respond to them?”

Exploratory questions such as “Please tell me more about it,” were also used to increase the depth of the interviews.

Each interviews lasted between 45 and 90 min, with an average of 60 min, depending on the willingness of the participants. The recorded interviews were then transcribed word by word. In order to ensure the validity of the obtained data, some of the transcripts of the interviews were returned to the participants to verify the accuracy of them by reviewing.

### Data analysis

Data were analyzed using Colaizzi’s seven stage method with MAXQDA10 software. *T*his process was as follows: Data transcription with details, understanding the depth of meanings, extracting important phrases, placing concepts in categories and clusters and then forming themes, integrating study results to describe the phenomenon, stating the essential structure of the phenomenon, and finally validating the findings [20]. Several strategies were also used to validate the findings. The researchers selected the participants by considering the maximum variation and their characteristics such as age, education, work experience, and place of employment. The researchers also tried to avoid their assumptions at all stages of data collection and analysis processes and spent enough time on conducting in-depth interviews and collecting and analyzing them to ensure an in-depth and accurate understanding of the data. As well, in order to increase the validity of the data, the whole data analysis process was reviewed and controlled by two members of the research team.

## Results

Twelve participants, including 4 gynecologists, 6 midwives working in hospitals and private offices, and 2 midwives working in health centers, were interviewed in this study. The age range of the participants was between 36 and 57 years old and their work experience ranged from 5 to 28 years. The complete characteristics of the participants included in this research are given in Table 1.

**Table 1:** Demographic characteristics of participants (*N* = 24)

Participants (No)	Age (years)	Work experience (years)	Profession	Place of employment
1	56	26	gynecologist	Hospitals and private offices
2	42	5	gynecologist	hospitals
3	55	10	gynecologist	hospitals and private offices
4	57	17	gynecologist	hospitals and private offices
5	45	25	midwife	hospital
6	40	15	midwife	hospital
7	45	18	midwife	hospital
8	45	12	midwife	hospital
9	36	5	midwife	hospital
10	45	17	midwife	hospital
11	50	25	midwife	Health center
12	40	13	midwife	Health center

Qualitative data analysis resulted in a total of 120 initial codes. Thereafter, different codes, based on their similarities and differences with each other and based on the homogeneity of the content, were grouped into the subcategories and then into the main categories.

At the end of this stage, 25 subcategories, 9 main categories, and 3 themes were obtained. The three themes extracted from the data analysis were as follows: “Fear of Disease”, “Burnout”, and “Lessons Learned from the COVID-19 Pandemic”, respectively, the details of which are described in Table 2.

**Table 2:** The main themes and extracted categories

Theme	Category	Sub-category
1. Fear of disease	Reducing maternal referrals for prenatal and postnatal care	Reduction in the number of prenatal care referrals in health centers
		Reduction in the number of referrals to hospital centers
		Reduction in the activity of private offices
	The preference of mothers to give birth in safe centers	Tendency to delivery in private centers
		The increased mothers’ referrals to non-hospital delivery centers
	concerns on maternity complications	Neglecting the complications of pregnancy and childbirth
		Attention to and focus on COVID-19
	Workplace insecurity	Unknown nature of the disease
		Asymptomatic carriers
		shortage of personal protective equipment (PPE)
2. Burnout	The increased workload	New roles
		Infection of employees and staff shortages
	The reduced family relationships	concern on transmitting the disease to the family
		Entrusting children to grandparents and staying away from them
	lack of motivational factors	Lack of encouraging supports
		Comparing their working conditions with other staff
		Lack of empathy of officials
3.Lessons learned by the COVID-19 Pandemic	Adaptation to new conditions	The increased knowledge about the disease to overcome conditions
		Enhanced team working among staff
		Inter team cooperation
	The need to strengthen and support maternity health services	Providing consulting services to staff
		Providing a platform for maternity care at home
		Using the capacity of telemedicine for maternity services
		The need for incentive processes for maternity care staff
		The need for performing rapid screening tests in birth centers

### Theme 1: “Fear of Disease”

This theme is an abstraction of the concepts of the following 4 main categories: “ Reducing maternal referrals for prenatal and postnatal care”, “ The preference of mothers to give birth in safe centers “, “ concerns on maternity complications “, and “ Workplace insecurity “ .

At the onset of the COVID-19 pandemic, fear of becoming infected as well as fear of transmitting COVID − 19 to the baby caused a reduction in the number of referrals of many pregnant mothers for prenatal care. Meanwhile, the number of pregnant mothers referred to hospitals and health centers which were introduced as COVID-19 referral centers had a much more significant reduction. In addition, many Private offices were deactivated in this regard.

“Some health centers were introduced as referral centers for COVID-19, which caused a sharp reduction in the number of pregnant women who were referred to those centers for prenatal care and the pregnant mothers were afraid of visiting these centers.” (P 11)

Considering the reduction in prenatal care, the possibility of pregnancy complications increased due to the neglect.

“In the second wave of the disease, some pregnant women referred to the hospital by manifesting some symptoms of high blood pressure. They had severe preeclampsia and needed magnesium sulfate, may be due to a lack of routine visits and blood pressure control in previous months because of fear of COVID-19.” (P 7)

“In some cases, the symptoms of embolism and bleeding during surgery were mistaken instead of COVID-19 symptoms, and the mother consequently died.” (P 2)

The participants of this study pointed out that, at the beginning of the COVID-19, the disease was highly unknown and lack of knowledge on the nature of the disease had caused confusion about how to deal with that.

On the one side, lack of knowledge of the health care provider about this disease and on the other side, lack of personal protective equipment, caused work stress in staff during the outbreak of this disease.

“There is not enough protective equipment. For example, the mask for the mother is not in the delivery package and some pregnant mothers go to the delivery with no mask, and afterward, there is no opportunity for her to buy a mask. So, we have to give a mask to the mother from our quota, or the mother mask is contaminated in labor or it gets wet and we have to give them our mask quota, while we do not have enough mask for our staff.” (P 9)

### Theme 2: “Burnout”

This theme was formed from the abstraction of the concepts of the following three main categories: “Increased workload “, “Reduced family relationships “and “lack of motivational factors” as well as its 7 sub-categories.

During the outbreak of the disease, the majority of health care workers contracted COVID-19 due to their occupational exposures. This consequently reduced the number of staff per shift and simultaneously increased the work responsibilities of other staff. This disease also led to the creation and addition of new roles and responsibilities for staff, which in turn increased their workload, fatigue, and burnout.

“The staff became very involved in this disease. About 80% of them became infected and we had a shortage of staff. The numbers of offs and staff per shift were less and we all suffered from a chronic exhaustion that did not go away.” (P 6)

According to the participants, fear of transmitting the disease to the family has reduced their family relationships. Moreover, some of them have been forced to leave their small children with their grandparents due to the closure of kindergartens. Thereafter, their lack of contact with their families has weakened their morale, which then made them extremely tired from working.

“We are suffering from a guilty conscience because our families may be affected by this disease. Inevitably, we further reduced our contact with our families.” (P 8)

All midwives participating in the study had complaints of burnout and lack of motivational factors in the workplace and pointed out the need for encouraging supports.

“Unfortunately, hospital managers do not understand the difficulty of our work. Furthermore, they do not consider any incentives for us and all their attentions are paid to those employees who are in the corona ward. While all healthcare providers in every sector are involved in corona hazards. ” (P 10)

### Third theme: “Lessons learned from the COVID-19 Pandemic”

This theme was formed from the abstraction of the concepts of the two main categories named as “adaptation to new conditions” and “ The need to strengthen and support maternity health services “ as well as its 8 sub-categories.

Over time, the nature of the disease was better known and global and national guidelines on maternity care in the context of the COVID-19 were gradually developed. The knowledge and skills of the maternity care staff in order to provide services to pregnant mothers increased and the fear from the unknown nature of the disease decreased and the health care staff gradually learned how to be adapted to this new critical condition.

“At first, we were afraid of symptomatic patients and reluctant to take care of them, but currently, we take care of them by following the related protocols and wearing personal protective equipment. This may be due to obtaining more knowledge on the disease and now we bacame adapted to these conditions.” (P 7)

Coronavirus has taught them that in critical situations, inter-group empathy and teamwork can solve many problems.

“In the current Coronavirus outbreak, we tried to collaborat with our colleagues . we tried to make midwives who had a chronic illness or had a small child have less contact with mothers with COVID-19, and other midwives on shifts cared the patients.” (P 5)

According to the opinion of the participants, due to the psychological stress caused by working conditions during the COVID-19 pandemic, the need to provide counseling services to them seems more necessary.

“I feel that all the medical staff are tired spiritually and physically. However, for hospital managers, this does not matter at all. I wish it was possible to hold some psychological counseling sessions for staff.” (P 9)

One of the concerns mentioned by the participants was the possibility of increasing maternal and fetal complications due to the reduced maternal referrals for prenatal care. Therefore, the need to provide a platform for midwifery services at home in Iran, as in many other countries, are more clear than ever. Accordingly, this provides essential maternity care in a safer and less stressful environment.

“I believe that during the COVID-19 pandemic, providing midwifery services at home can consequently reduce the risk of transmitting this disease to the pregnant mother. ” (P 4)

Notably, one of the main concerns of physicians and midwives working in the delivery room is the presence of asymptomatic positive carriers. In their opinion, the need for rapid screening tests in delivery room is necessary that can be useful in reducing the transmission of the disease to staff and mothers attending the delivery.

“Pregnant mothers who go to the hospital for delivery have no screening for COVID-19, unless they manifest some of its symptoms. Additionally, screening tests are time consuming and in the case of emergency deliveries, there is not enough time to perform these tests in the delivery room.” (P 9)

One of the common and positive experiences of physicians and midwives participating in the study was the use of telemedicine capacity to provide maternity care during the current COVID-19 pandemic. Establishing telephone communication with mothers to follow up pregnancy and childbirth care, forming virtual groups and making video calls to mothers, answering their questions, and providing maternity care virtually are the examples of the efficient usage of such facilities. The use of telemedicine for maternity care reduced the number of referrals of pregnant women for care and it also was very useful in relieving their anxiety and helped in breaking the chain of disease’s transmission.

“ I set up a virtual channel with midwives to follow up those pregnant mothers who came to my office. With the help of virtual facilities, we provided training classes for mothers for one hour a day and then guide them. In this way, we were able to reduce the stress and depression of pregnant mothers in the epidemic of COVID-19.” (P 1)

## Discussion

This was the first study conducted in Iran aimed to understand the experiences of health care providers from maternity care during the current COVID-19 pandemic. The results of this study show that at the onset of the COVID-19 pandemic, fear of becoming infected along with fear of transmitting COVID-19 to the baby caused a reduction in the referral of many pregnant mothers for prenatal care. Most mothers were reluctant to go to health centers and hospitals, which were introduced as referral centers of COVID-19. They were looking for a safe place to receive maternity care. Accordingly, the desire of mothers also increased to give birth in private hospitals, because there is no home birth in Iran. This finding falls in line with the results from Gutschow et al. [21] in the United States, healthy pregnant women increasingly seek to give birth out-of-hospital. Also, the results of a study conducted in 81 countries were in line with our results [5, 21].

The reduction in the number of referrals for maternity care, along with too much focusing on COVID-19 and neglecting the possible complications caused by pregnancy and childbirth, have led maternity care providers to be concerned about the possible increase in both maternal and neonatal morbidity and mortality. In a study by Karavadra et al. (2020), pregnant mothers reported that prenatal visits were not as complete as before, and they were concerned that some aspects of their prenatal care may be missed [16]. The results of other studies in this field also confirmed the concern that changes in the provision of standard maternity care may lead to poor outcomes related to maternal and neonatal health statuses [5, 22–25]. Therefore, the long-term effects of COVID - 19 on prenatal care should not be ignored and more attention should be paid to proposing quality maternity services, especially for high-risk pregnant mothers during the current epidemic.

Additionally, based on the opinions of the majority of the study participants, to ensure quality maternity care to mothers during the current COVID-19 pandemic and other possible future critical situations, it is necessary to use the midwifery potential in providing comprehensive maternity care at home.

The COVID-19 pandemic reveals preexisting dysfunctionalities in maternity care. We know that hospitals are the main source of disease transmission, and yet, most maternity care and deliveries are done in the hospitals. It is now clear that a decentralized approach to maternity care, which includes the use of a variety of facilities such as maternity care centers and midwifery home care for low-risk women with access to higher-level facilities where needed, results in better outcomes and is more cost-effective and woman-centered. Accordingly, flexible models of midwifery care can be used to ensure quality maternity care in the context of COVID − 19 pandemic as well as other similar critical situations in the future [21, 26]. We hope that providers and policymakers will use the lessons learned from the COVID-19 pandemic to make fundamental changes in midwifery services in Iran and support and establish a decentralized maternity care model that integrates midwives with obstetricians and community birth providers with hospitals.

Utilizing the capacity of telemedicine as well as forming virtual groups and channels can increase the relationship between care providers and mothers, which decrease the possible consequences of reducing maternal visits during the current COVID-19 pandemic. However, this new model of care does not apply to all mothers and in all regions of the country, and the lack of access to adequate internet connectivity in some places is known as one of the obstacles to the use of telemedicine [5].

According to the findings of the present study, at the beginning of the COVID-19 outbreak, the unknown nature of the disease, rapid changes in guidelines, and lack of protective equipment caused confusion among health care providers as well as fear of the disease. The results of our study are in line with the results of the study by Liu et al. (2020) and Semman et al. (2020). In this regard, they stated that health care providers have experienced persistent fear in the epidemic due to the contagious nature of this virus, unknown routes of transmission, close contact with patients, lack of protection facilities, and the ongoing illness of their colleagues [5, 14]. Kockin et al. (2021) believe that these psychological reactions, are normal reactions to crisis and experiences from crises caused by previous infectious diseases also have shown that in the first phase of the crisis, the health staff had fears and negative emotions [27].

Maternity ward staff face special challenges such as close relationship with mother and baby, providing ongoing and necessary support for women in labor and delivery, and limited childbirth room space that put maternity ward staff at higher risk for contracting COVID-19 [17].

Of note, one of the most important concerns mentioned by maternity care providers was the presence of asymptomatic carriers and the taboo of expressing their illness. Special working conditions in the delivery room such as lack of sufficient time to screen pregnant mothers in the delivery room and the impossibility of having social distancing during delivery and inability of mothers to tolerate the mask during delivery, currently increase the risk of transmitting the disease to staff in the delivery ward. In addition, the lack of personal protection facilities in maternity care units has increased their fear experience.

In many European countries, midwives want to receive personal protective equipment and safe working conditions, to be able to provide safe and respectful care for pregnant mothers [28]. The lack of facilities for screening the disease put the safety of the workforce and mothers in danger [5]. According to the RCOG recommendation, it is better to equip maternity wards with rapid screening tests in the near future [29].

One of the main themes of the findings of this research was the experience of burnout. Notably, almost all maternal health care providers are suffering from exhaustion and work-related stress during the present COVID-19 pandemic. Accordingly, most of them contracted COVID-19 due to their occupational exposures. This has consequently reduced the number of staff per shift and simultaneously increased the work responsibilities of other staff and caused unpredictable changes in their work schedule. Coronavirus disease also led to the creation of new roles and responsibilities for staff, which in turn increased their workload, fatigue, and burnout. In addition, the study participants reported that they reduced their family relationships due to the fear of transmitting the disease to their families, which made them feel lonely. Consistent with our study results, Fathi et al. (2020) stated that medical staff, due to the fear of transmitting COVID-19 to others, have reduced or cut off their communication with those around them and the majority of them experienced loneliness and lack of social support [30].

Work-related stress during the current COVID-19 pandemic, on the one hand, and the lack of incentives and motivation factors in the workplace, on the other hand, increase the burnout of maternal health care providers.

Alizadeh et al. [31] stated that the three main psychological needs, namely autonomy, competence, and communication must be satisfied to ensure the mental health of people in their workplace. in the corona outbreak, these needs for health care providers are disrupted by the cancellation of vacations, extra and unintended shifts, lack of facilities in the workplace, the unknown nature of the disease and decreased social and family relationships. Burnout is likely to increase psychological distress and decrease responsibility in the workplace [31].

Burnout may reduce some of their communication skills such as empathy with pregnant mothers, and then lead them to respond inappropriately to the needs and concerns of women receiving maternity care services [32].

Leaders and managers can play an important role in reducing employee burnout. In order to reduce psychological problems of healthcare staff in the COVID-19 Pandemic, strategies such as Increasing positive interaction between managers and employees, increasing facilities and resources,, fair distribution of duties and resources, adequate and timely payments, and Spiritual and financial incentives are recommended [31]. Regular assessments of their mental health and providing access to free psychological counseling services are very useful in this regard. Managers should also be aware of the needs of maternal health care providers and then try to meet them. Encouragement and providing spiritual support to them in these situations could help in reducing their burnout and could also be effective in providing quality services to women.

According to findings, with the passage of time, the dominance of health care staff in providing services to pregnant mothers has increased, the fear of the unknown nature of this disease has reduced, and health care staff have gradually learned how to be adapted to this new critical condition. They acknowledged the importance of teamwork, empathy, and intergroup collaboration to be better adapted to new changes in their workplace. In this way, the role of individuals and teams must be defined, mutual trust and respectful environments and a sense of belonging established, and efficient communication maintained [33].

Gutschow et al. (2021) believe that dialogue between all kinds of maternity care providers (midwives and obstetricians) promotes evidence-based care and it is better for obstetricians to consider midwives as colleagues rather than subordinates personnel [21, 26]. In the context of the COVID-19 pandemic, promoting inter-professional collaboration should be known as a priority to ensure providing efficient and quality care [33].

## Limitations

In this study, the combination of the participants (8 midwives and 4 physicians) was unequal. However, the authors did not aim to compare experiences between midwives and physicians, and since the number of midwives working in maternity care units is more than that of physicians, so the combination of the samples seems appropriate.

## Conclusions

The study results show that maternal health care providers experienced more emotional and psychological stress and work challenges during the current COVID-19 pandemic. Given the negative and undeniable effects of these tensions on the provision of maternity healh care, it is necessary that health service officials should be prepared before the crisis in order to provide the necessary and sufficient equipment for improving the working conditions of caregivers of maternity units.

## Data Availability

The datasets used and analyzed during the current study are available from the corresponding author on reasonable request.

## Abbreviation

PPE: Personal protective equipment
